# Role of Lactic Acid Bacteria in Food Preservation and Safety

**DOI:** 10.3390/foods11091283

**Published:** 2022-04-28

**Authors:** Agnieszka Zapaśnik, Barbara Sokołowska, Marcin Bryła

**Affiliations:** 1 Department of Microbiology, Prof. Waclaw Dabrowski Institute of Agricultural and Food Biotechnology—State Research Institute, Rakowiecka 36, 02-532 Warsaw, Poland; agnieszka.zapasnik@ibprs.pl; 2 Department of Food Safety and Chemical Analysis, Prof. Waclaw Dabrowski Institute of Agricultural and Food Biotechnology—State Research Institute, Rakowiecka 36, 02-532 Warsaw, Poland; marcin.bryla@ibprs.pl

**Keywords:** lactic acid bacteria, lactic acid fermentation, mycotoxins, foodborne pathogens

## Abstract

Fermentation of various food stuffs by lactic acid bacteria is one of the oldest forms of food biopreservation. Bacterial antagonism has been recognized for over a century, but in recent years, this phenomenon has received more scientific attention, particularly in the use of various strains of lactic acid bacteria (LAB). Certain strains of LAB demonstrated antimicrobial activity against foodborne pathogens, including bacteria, yeast and filamentous fungi. Furthermore, in recent years, many authors proved that lactic acid bacteria have the ability to neutralize mycotoxin produced by the last group. Antimicrobial activity of lactic acid bacteria is mainly based on the production of metabolites such as lactic acid, organic acids, hydroperoxide and bacteriocins. In addition, some research suggests other mechanisms of antimicrobial activity of LAB against pathogens as well as their toxic metabolites. These properties are very important because of the future possibility to exchange chemical and physical methods of preservation with a biological method based on the lactic acid bacteria and their metabolites. Biopreservation is defined as the extension of shelf life and the increase in food safety by use of controlled microorganisms or their metabolites. This biological method may determine the alternative for the usage of chemical preservatives. In this study, the possibilities of the use of lactic acid bacteria against foodborne pathogens is provided. Our aim is to yield knowledge about lactic acid fermentation and the activity of lactic acid bacteria against pathogenic microorganisms. In addition, we would like to introduce actual information about health aspects associated with the consumption of fermented products, including probiotics.

## 1. Introduction

Fermentation technologies are of considerable significance for the food industry because they enable the preservation of food products and prolong their shelf-life while at the same time providing them with the desired sensory properties. Moreover, they have a favorable impact on the health-promoting value of food due to the presence of probiotic microorganisms and increasing nutrients in the product. In addition, they can increase microbial safety [1,2]. During a fermentation process, the development of undesirable microflora and the formation of unfavorable compounds is inhibited by metabolites of the microorganisms taking part in fermentation [3]. This is a highly desirable phenomenon, because it is linked with the possibility of reducing the addition of chemical preservatives to foods. 

Fermentation processes are the oldest biotechnological techniques used in food production, and they are currently among the primary processes used in the food industry. Fermented products, including bread, cheese, soy sauce, wine, beer, vinegar and many others, have been present in the human diet since the beginnings of civilization development. Traditionally, fermentation was conducted spontaneously, which resulted in low efficiency and variable quality of the final product. Presently, selected starter cultures are used in the conditions of industrial production. On the other hand, regional and craft products are often still based on spontaneous fermentation [2,4]. 

Not all freshly fermented products are suitable for instant consumption, because certain biochemical changes require time. The maturation process contributes to achieving stability and enhancement of the sensory quality of products due to the formation of specific flavoring compounds, including diacetyl, carboxylic acids, aldehydes, ketones and esters. These characteristics contribute to increased acceptability by consumers, who, apart from the health-promoting values, pay attention to the sensory attractiveness of fermented foods [1,2,3,5]. Fermentation consists in the metabolism of carbohydrates, proteins and fats under the influence of specific microorganisms, including yeasts, bacteria and filamentous fungi. In order to set a determined direction for the fermentation process, specific substrates and microorganism strains are used [3]. Depending on the selected substrates and microbial cultures, the process itself may assume the form of lactic, alcoholic, propionic, citric, butyric, methanol, mannitol or acetic fermentation [6]. 

Biopreservation, understood as a biological method for preserving foods with the use of microorganisms and their metabolites, has gained significant interest in recent years due to the increased awareness of consumers regarding chemical preservatives and their negative impact on health [7,8]. The most important chemical preservatives and their effects on human health are described in Table 1. 

Microorganisms used for the purpose of natural preservation should meet a range of requirements, including safety of use, the production of non-toxic metabolites, maintaining high activity during storage and the absence of a negative impact on the product’s sensory properties [12]. LAB are of particular importance in biopreservation processes due to the wide spectrum of their activity against the development of unfavorable microflora [13]. The aim of the study was to yield the available knowledge on the importance of the lactic acid fermentation process in enhancing food safety and the activity of LAB against food pathogens, including bacteria, yeast and filamentous fungi. In addition, we would like to highlight the health benefits associated with the consumption of fermented foods with LAB. 

## 2. LAB

Lactic fermentation is used, inter alia, for milk acidification and thus the production of fermented dairy products, such as yogurts, cheese, butter, sour cream, etc. [12]. Moreover, the process is responsible for the formation and stabilization of vegetable silage and sourdough and is used for cold cut maturation [14]. Fermentation occurs with the participation of homo- and heterofermentative LAB. Predominant cultures used in the processes of lactic fermentation are bacteria classified in the genus *Lactococus*, *Streptococcus*, *Lactobacillus*, *Leuconostoc*, *Pediococcus*, *Weisella* and *Bifidobacterium*. 

Homofermentation consists in the metabolism of disaccharides by select LAB strains to almost pure lactic acid. Heterofermentation is a slightly different process, where, as a result of lactose decomposition, ethyl alcohol, carbon dioxide, hydrogen peroxide, diacetyl, acetoin and acetic aldehyde are formed apart from lactic acid [14,15,16]. 

From the process standpoint, lactic fermentation is the easiest to conduct. The decrease of natural pH below 4.0 that occurs during the process does not have a negative effect on the efficiency of biochemistry, due to the dominance of LAB, capable of adapting to the low pH of the environment [17].

LAB are gram-positive, non-spore-forming and incapable of producing catalase bacilli and cocci. They are classified among relative or obligatory anaerobes, and they tolerate the acidic pH of the environment [18,19,20]. In April 2020, in the official register of the International Journal of Systematic and Evolutionary Microbiology, new nomenclature of *Lactobacillus* and *Leuconostoc* bacteria was published [21]. The purpose of that change was to systematize bacteria, which, due to high diversity, required correct classification. Modern methods of molecular biology enabled the introduction of expanded taxonomy for the genus [22]. In the present article, species names of the microorganisms will be used in accordance with the spelling used in the given source article.

LAB are generally considered to be safe (GRAS) and are widely used in the food industry; moreover, they form the natural microflora of human intestines [23,24]. In the context of biopreservation, LAB play a very important role due to the fact that, during the growth and fermentation process, they produce a range of metabolites with antimicrobial action, which include hydrogen peroxide, lactic acid, acetic acid and low molecular weight substances (diacetyl, fatty acids, reuterin, reutericyclin), antifungal compounds (phenyl lactate, propionate, hydroxyphenyl lactate) and bacteriocins [25]. 

## 3. Bacteriocins

The bacteriocins group mainly consists of generally thermostable protein substances featuring antimicrobial properties. It is assumed that the effect of bacteriocins is based on the binding of phosphate residues present on cell membranes of the target cells, creating pores and the activation of autolysin that degrades the bacterial cell walls [26]. Bacteriocins belong to the diverse group of cationic and hydrophobic peptides built of 20–60 amino acids. Furthermore, their synthesis is based on ribosomal machinery. Bacteriocins encoding genes are located in operons in plasmids, chromosome and other genetic organelles [27]. One of the most important attributes of bacteriocins is their activity against other bacteria, fungi, viruses, parasites and natural structures such as biofilms [27,28]. Alvarez-Sieiro et al. [29] proposed the classification of bacteriocins produced by LAB based on three main classes. The class I includes modified, heat stable and low molecular weight peptides consisting of unusual amino acids such as lanthionine. The class II consists of unmodified thermostable, low molecular weight bacteriocins. The last class is the only group of thermolabile and high molecular weight substances [30]. Their activity takes different directions, such as bactericidal or bacteriostatic effects on species related with the producing strain. The environmental factor stimulates the production of bacteriocins, including nutrient availability, the density of the bacterial cell, acetic acid and signal peptides’ presence. The mechanism of their activity is based on their primary structure. Some bacteriocins have the ability to enter the cytoplasm of other bacteria and affect their gene expression and the synthesis of protein. On the other hand, some of them can exert their activity on the cytoplasmic membrane, contributing to cell lysis by releasing vital compounds of susceptible microorganisms [27]. The significant advantage of bacteriocins is their activity against opportunistic and pathogenic bacteria, including antibiotic-resistant strains. Furthermore, several bacteriocins show their synergy with antibiotics, contributing to reducing concentration and negative side effects. Their synergistic activity with other biomolecules such as citric acid and nisin against *Listeria monocytogenes* and *Staphylococcus aureus* is well known [31]. However, it is important to notice that the mentioned bacteria can develop a resistance to bacteriocins, but it is minimal compared to the conventional antibiotics’ resistance [31]. Bacteriocins constitute a group of highly attractive substances for the food industry due to their non-toxicity towards human organisms, thermal stability, protein nature and antagonistic effect towards the majority of Gram-positive microorganisms [13,32]. In the present time, the application of bacteriocins produced by LAB is limited in the food industry. Only the lantibiotic nisin (E234) and pediocin PA-1/Ac H are commercialized in the food supply chain as preservative agents [30].

## 4. Health-Promoting Values of Products Fermented with LAB

Numerous studies indicate that lactic fermentation has a positive effect on the nutritional value and increased digestibility of raw materials subject to the process. The acidic nature of fermentation increases the activity of enzymes produced by specific microorganisms, including amylases, proteases, lipases and phytases, thus modifying the raw material through the hydrolysis of polysaccharides, proteins and fat [33,34]. Through the increasing activity of microbial enzymes, the number of anti-nutritive compounds, such as phytic acid and tannins is reduced. These compounds negatively affect the bioavailability of minerals, including iron, proteins and simple sugars. Moreover, the number of vitamins in the product is also increased due to the fermentation process and the activity of specified microorganisms [1,35].

The health-promoting properties of LAB are based mainly on the increase in the bioavailability of nutrients, antioxidant activity, the biosynthesis of vitamins and the degradation of antinutritional ingredients. The antioxidant activity of LAB is linked to their capability to transform phenolic acids to biologically active forms through the decarboxylation of phenolic acid and the effect of reductases and hydrolases. This capability is of considerable significance in the case of plant material fermentation [36]. In the context of the increasing nutritional value of foods, LAB may increase the content or bioavailability of vitamins. 

Numerous authors have conducted experiments aiming at testing the effect of LAB on the content of vitamin C. The results thus far are not homogeneous; however, some studies point to a positive effect of LAB on the content of ascorbic acid. Kazimierczak et al. [37] determined that a spontaneously fermented beetroot juice was characterized by higher vitamin C content relative to juice not subject to fermentation. Studies showing reduced vitamin C content during fermentation can be explained by the fact that, with fermentation time, ascorbic oxidase activity may increase due to the fermenting microflora [38]. Sharma et al. [38], in their research, show that the content of vitamin C in the natural fermented Indian beverage Kanji increased during the fermentation process and was stable for the next 40 days of storage, but after that time, the content gradually reduced. 

LAB and Bifidobacteria have the capacity to transform individual diet components into group B vitamins and vitamin K, where the first group of vitamins plays a fundamental role in the normal function of human organisms. *Lactibacillus reuteri*, *Lactiplantibacillus plantarum* (*Lactobacillus plantarum*), *Lactobacillus acidophilus* and *Bifidobacterium longum* deserve special attention in the context of the biosynthesis of group B vitamins [35,36,39]. Vitamin K is well known due to its role in the production of blood clotting proteins. It is associated with the significant role of vitamin K as a cofactor for the formation of y-carboxyglutamic acid (Gla) in proteins, which bind calcium ions and participate in the blood coagulation and calcification of tissue [40]. Vitamin K is a fat-soluble chemical compound, which occurs in two main forms: K1 (phylloquinone) in plants and K2 (menaquinones (MK)) in animals and bacteria. The main source of vitamin K intake is vegetables (80–90% dietary intake), but the absorption is about 5–10%. In comparison, the absorption of vitamin K (MK) from dairy products may achieve almost 100% [41]. The study of Morishita et al. [40] confirms the ability of LAB to produce a meaningful amount of vitamin K and suggests the possibility of usage selected strains as a starter culture for the production of fermented foodstuffs or dietary supplements.

Oxidative damage is a global concern because of its negative impact on human health. It is associated with several diseases such as cancer, cirrhosis, inflammatory diseases and atherosclerosis [42]. The antioxidative and anticarcinogenic potential of LAB is a significant subject due to their possible usefulness for preventing cancer diseases. According to the study of Shehata et al. [42] there is correlation between high antioxidant activity and the anticarcinogenic properties of bacterial lysate. The study found that two of the tested strains (*Streptococcus thermophilus* BLM 58 and *Pediococcus acidilactici* ATTC 8042) had the strongest antioxidative effect. Various studies show the high anticarcinogenic activity of LAB [43,44]. Pourramezan et al. [45] investigated the anticancer, antioxidant and apoptotic properties of some strains of Lactobacillus isolated from traditional doogh samples. The tested strain Lactobacillus AG12a shows high anticarcinogenic and antioxidative activity in vitro. However, the studies should be tested in vivo in order to validate these findings. Vamanu et al. [46] suggested that including probiotics in a daily diet may decrease the possibility of carcinogenesis of the colon due to the inactivation of carcinogenic compounds, the stimulation of immune system and the reduction in the activity of enzymes in the digestive system, which may contribute to the conversion of procarcinogens into carcinogens. 

Moreover, certain LAB strains exhibit probiotic properties. In accordance with the WHO (World Health Organization) definition, probiotics are live organisms that, when provided at a specific dose, have a positive effect on the host’s organism. Probiotic microorganisms must also fulfill a range of requirements, i.e., they should be isolated from human organisms, exhibit resistance to difficult conditions of the gastrointestinal tract (low pH, presence of gastric acid) and they must be characterized by high adhesion to the intestinal epithelium and a complete lack of virulence [47]. Probiotic properties should be assigned to a specific strain and not genus or species [48]. Probiotic bacteria exhibit a favorable impact on reducing blood cholesterol levels and its metabolism, and, in addition, through the host organism colonization, they may contribute to reducing the risk of carcinogenesis and the stimulation of the immune system [47]. Probiotics may also play a significant role in gastrologic problems through the inhibition of pathogenic microorganism adhesion to the intestinal epithelium and the synthesis of antibacterial substances, i.e., bacteriocin or organic acids [49]. Furthermore, they participate in the biosynthesis of vitamins, and the metabolites produced by them regulate the homeostasis of the gastrointestinal system [50,51]. Table 2 presents characteristic products obtained as a result of lactic fermentation, listing dominant and collaborating microflora.

## 5. Use of LAB against Foodborne Bacterial Pathogens 

Foodborne pathogens occurring in food manufacturing and provoking various diseases related to the consumption of contaminated products constitute a critical point in the food industry. Scientists continue to search for innovative and safe methods of food preservation, including the lactic fermentation process with LAB as a safe method for human health [68]. Many authors demonstrated the inhibiting effect of LAB towards the development of foodborne pathogens, such as *Salmonella* spp. [69], *Listeria monocytogenes* [70] and *Escherichia coli* [71].

During their growth and fermentation process, LAB produce a range of metabolites with antimicrobial effects, the action of which is based on the destabilization of the membrane, the inhibition of the synthesis of cell wall enzymes, the interference of proton gradients and the induction of the formation of reactive oxygen species, thus increasing oxidative stress within the cell [72]. The majority of scientific reports suggest that the action against the pathogenic microflora is mainly based on the formation of conditions difficult for their development due to pH reduction under the lactic acid produced by them at considerable amounts. The remaining organic acids formed as a result of fermentation, i.e., acetic and propionic acid, exhibit antagonistic effects against the development of bacteria, yeasts and filamentous fungi; however, the synthesized amounts of these acids are not significant [73,74]. The pH reduction caused by the presence of organic acid produced by LAB efficiently inhibits the development of *Salmonella* spp. bacteria, which are intolerant of low pH, and their optimal growth remains in the 4.0–9.0 range [68]. Choi et al. [75] investigated the antagonistic activity of LAB isolated from naturally fermented kimchi against selected pathogenic strains, including *E. coli O157:H7*, *Salmonella typhimurium*, *Staphylococcus aureus* and *Salmonella enteritidis*. The experiment demonstrated the inhibiting effect of the used strains on the development of pathogens; however, it was not linked to the activity of bacteriocins, hydrogen peroxide or fatty acids. The key compound reducing the quantity of pathogenic microorganisms was lactic acid. These results were confirmed in other studies, which determined that lactic acid is the predominant factor contributing to the inhibition of undesirable microflora. The study of Stanojevic-Nikolic et al. [76] assessed the effect of lactic acid on the development of pathogens. It was demonstrated that lactic acid is more efficient towards Gram-positive than Gram-negative bacteria, and, with the increase in the acid, the efficacy at which the development of pathogenic microflora is inhibited increases.

Bacteriocins also contribute to the inhibition of microorganism development. In the study of [77], the efficacy of the action of nisin synthesized by *Lactobacillus bulgaricus* and *Streptococcus pyogenes* strains towards pathogens, i.e., *Bacillus subtilis*, *Pseudomonas aeruginosa*, *Serratia marcescens* and *Staphylococcus aureus*, was assessed. The effect of nisin was more pronounced towards Gram-negative bacteria, which is linked to the structure of their cellular membrane. The study of Scatassa et al. [78] showed that the production of cheese with the use of a mixture of *Lacticaseibacillus rhamnosus* (*Lactobacillus rhamnosus*), *Lactococcus lactis* and *Enterococcus faecium* may result in the inhibition of *Listeria monocytogenes* growth through the secretion of bacteriocin-like substances. In another study, Wang et al. [79] observed an inhibiting effect of metabolites produced by LAB on the development of *Bacillus licheniformis* isolated from milk powder. This experiment indicated that, under controlled pH conditions, *Lactiplantbacillus plantarum* (*Lactobacillus plantarum*) had an inhibitory effect on the growth of cells and biofilm production by *B. licheniformis*. The efficacy of *L. plantarum* in the inhibition of biofilm formation was confirmed on matrices, i.e., glass and steel. This study is of particular importance for the dairy industry, where efficient methods for the removal of bacterial biofilms are searched for. *Salmonella* bacteria are capable of adhering and forming biofilms on glass, rubber and metallic surfaces. Biofilms contribute to food spoilage and constitute the critical point in production facilities due to their resistance to cleaning and disinfection. They can be formed on any type of surface, including metal, plastic, wood, glass and stainless steel [80]. Todhanakasem and Ketbumrung, [80] assessed the efficacy of the application of LAB to control the formation of biofilms by *Salmonella enterica* ssp. *enterica* and *B. cereus*, *E. coli* bacteria. LAB isolated from fermented food turned out to be efficient in inhibiting the proliferation of bacterial pathogen cells and biofilm formation. It is necessary to conduct further research on the efficacy of LAB under in situ conditions and to assess their application in the food chain. 

## 6. Use of LAB against Yeast

Traditionally, yeasts are known as the most important microorganisms due their role in the production of bread, alcoholic beverages and dairy products, as well as their role as an ethanol for fuel, extracts and pigments or biochemicals for the pharmaceutical industry. However, yeast contribute to the spoilage of food and beverages. The negative role of yeasts is associated with their ability to grow in low temperatures and pH values as well as their resistance towards physico-chemical stress [81]. The occurrence of unwanted yeast such as *Kloeckera apiculata*, *Brettanomyces bruxellensis*, *Rhodotorula mucilaginosa*, *Schizosaccharomyces pombe*, *Candida krusei*, *Candida parapsilosis*, *Debaryomyces hansenii*, *Pichia membranaefaciens* and *Zygosaccharomyces bailii* may contribute to problems with the quality and safety of products [82]. Yeasts can form undesirable microflora of fermented products and of the production environment. The cause of yeast contamination in the food chain may be the production facility itself due to the inappropriate hygiene system that can favor the biofilm formation on technological surfaces. It is an issue correlated with aerosols and overspray during sanitation. The biofilm formation by some species of yeast may occur as an important issue during food processing due to the significantly more complicated method of removal compared to planktonic cells [82]. The control of yeast is essential in the alcoholic beverages industry. Due to the high cost of substrate, alcoholic fermentation is processed without the previous sterilization of molasses feeding must or sugar cane, which causes the development of wild Saccharomyces cerevisiae strains as well as other yeast contaminants. It is a problem in the alcoholic beverages industry due to low productivity and operational issues. Species like *Candida tropicalis*, *Dekkera bruxellensis* or *Pichia galeiformis* may constitute a main determinant of decreasing the efficiency of alcoholic fermentation [83].

Of particular interest are the genera *Candida*, *Yarrowia* and *Meyerozyma* [84]. Yeasts of the genus *Candida* are microorganisms naturally inhabiting animal organisms, including the skin and mucous membranes. The infection is caused due to the overgrowth of *Candida* microflora, particularly in the case of lowered organism immunity or susceptibility to fungal infections [85]. *Yarrowia* and *Meyerozyma* yeasts play a significant role in the food supply chain as undesirable microflora contributing to the reduced organoleptic and microbiological quality of silage. Numerous authors have attributed the capability to neutralize or inhibit the development of pathogenic yeasts to LAB. 

The study of Coton et al. [86] assessed the capabilities of selected strains of *Leuconostoc*, *Lactobacillus* and *Propionibacterium* bacteria to inhibit yeast growth. It was demonstrated that the genus *Lactobacillus* was characterized by higher antimicrobial activity towards selected strains than *Lactococcus*. It was noted that *Yarrowia* and *Galactomyces* geotrichum yeasts exhibit the highest resistance towards the activity of LAB. According to the study of Yepez et al. [87], *Lactiplantibacillus plantarum* M5MA1(*Lactobacillus plantarum* M5MA1) turned out to be the most efficient strain, exhibiting antagonistic effects against i.a. *Meyerozyma guilliermondii*. This strain was described as a potential candidate posing an alternative for chemical preservatives. Bacteriocins also play a significant role in inhibiting the development of pathogenic yeasts. An example can be acidophilin, produced by *Lactobacillus bulgaricus* strain, exhibiting an efficient impact on *Candida albicans* [85]. 

## 7. Use of LAB against Filamentous Fungi

Filamentous fungi pose a serious problem in both the food industry and agriculture in general. They cause the contamination of food, feeds and crop diseases, contributing to serious economic loss [88]. Moreover, they are capable of the biosynthesis of toxic secondary metabolites, commonly known as mycotoxins. Some of them have proven to have a carcinogenic (fumonisin B1, aflatoxin B1, ochratoxin A), mutagenic (aflatoxins, fumonisins, ochratoxin A, toxin T-2), teratogenic (patulin, aflatoxin B1, ochratoxin A), estrogenic (zearalenone), nephrotoxic and hepatotoxic (aflatoxins, patulin) effect [89]. 

Numerous authors have been able to provide evidence for filamentous fungi development inhibition in fermented food as a result of the effect of LAB activity [90,91]. The mechanism of LAB activity against the development of filamentous fungi is mainly based on the action of their metabolites, which contribute to the deteriorated integrity of the cell membrane and the absorption of amino acids by fungi [92]. In the study of Yepez et al. [85], it was determined that isolated LAB strains originating from traditionally fermented vegetables (tocosh, chicha) exhibited efficacy in the inhibition of toxicogenic and non-toxicogenic strains of filamentous fungi of genera *Aspergillus*, *Fusarium* and *Penicillium*. Sadeghi et al. [93] assessed the antimicrobial activity of *Pediococcus pentosaceus* strain isolated from barley sourdough starter. Statistically significant efficiency of its action towards *Aspergillus niger* and *Aspergillus flavus* strains was demonstrated.

The majority of literature data on the capability of selected LAB strains for the inhibition of filamentous fungi growth present in vitro tests with the use of de Man, Rogosa and Sharpe (MRS) agar medium, which is selective for these bacteria. The composition of the medium is highly favorable for the development of LAB, and it probably induces their strong antimicrobial properties against filamentous fungi. However, it is often the case that, in in situ tests, the antimicrobial activity of LAB decreases or ceases completely [94]. Le Lay et al. [95] compared the activity of LAB and *Propionibacterium* under in vitro conditions (MRS agar medium) and in situ conditions (bakers’ wares). A marked difference in the antimicrobial activity of LAB and *Propionibacterium* in in vitro and in situ tests was observed. Under in situ conditions, only 12 (2 Propionibacterium) out of 69 strains exhibited antimicrobial activity towards filamentous fungi. In comparison, under in vitro conditions, out of 320 strains used, 103 showed high antimicrobial activity (53 out of 270 LAB strains; 49 out of 50 *Propionibacterium* strains). Table 3 presents examples of applications of specific LAB strains limiting the development of filamentous fungi and yeasts.

## 8. Use of LAB against Mycotoxins

The main source for mycotoxins is cereals and their products, but they can also be found in vegetables and fruit [108,109]. Their presence has been confirmed in products from animals fed with contaminated feed, such as milk or meat [109]. Many attempts have been made to either eliminate or reduce the level of contamination of crops with mycotoxins with physical (thermal processes) and chemical methods (acids, bases, oxidative and reducing compounds) [110]. However, such methods are associated with the risk of deteriorated health safety and reduced nutritional value. That is why, in recent years, scientists have turned to the possibility of using antagonistic microorganisms to detoxify cereals and yeasts. Numerous authors point to a high efficiency of LAB in neutralizing mycotoxins from the matrix, from small amounts to even their complete removal [111,112,113,114]. The mechanisms of detoxification are mainly based on biotransformation, biobsorption and bioadhesion [102]. 

Biotransformation aims primarily at the transformation of the given substance to its non-toxic or less toxic variant by means of changes occurring during the fermentation process and the activity of microorganisms and their metabolites [115]. Biabsorption is a technique utilizing the capabilities of selected microorganisms to absorb toxins to the inside of the cell. Unfortunately, the process is often reversible; thus, it has limited possibilities of being applied in the food industry. Bioadhesion consists in binding mycotoxins with the cell wall of the inactivated microorganisms [115]. 

A high concentration of mycotoxins in food has a negative impact on the capacity of antagonistic microflora for efficient action, which results in a prolonged time of adaptation to difficult conditions [116]. Fermentation also contributes to the reduced concentration of mycotoxins in raw material, which is directly linked to the presence of microorganisms involved in the process. An example here can be the reduction of Aflatoxin M1 in milk subject to fermentation during kefir or yogurt production [117,118]. The process of detoxification by lactic strains is highly rapid because the concentration of mycotoxins is reduced severalfold in the first 24 h of contact between the bacteria and the toxin. Extending the process does not appear to affect the increased efficiency of densification, and it even may contribute to the re-release of the substances to the environment, which is linked to the reversibility of the binding process [119]. The rate at which toxins are neutralized by LAB is also strictly linked to the growth conditions, including pH, cell concentration and the presence of nutrients and compounds inhibiting the growth of LAB [119]. The study of Zhou et al. [120] suggests the possibility of the degradation of mycotoxins by the substances released by LAB to their environment. In the study of Król et al. [121], the possibility of zearalenone neutralization by the selected LAB strains *Lactococcus lactis* and *Bifidobacterium* was considered, and the study focused on the antagonistic mode of action of these strains towards filamentous fungi. It was observed that toxin biosorption by *L. lactis* can be divided into two stages. The first one is characterized by a rapid decrease in zearalenone concentration by almost 90% in the sample, whereas in the second stage, the process slowed down and only 7% was bound. The neutralization of zearalenone by *Bifidobacterium* also takes place in two stages and is characterized by a similar course as in *L. lactis*. 

Fuchs et al. [23] tested the possibility of the detoxification of patulin and ochratoxin A with LAB. In the case of patulin, the best effect was obtained with a *Bifidobacterium animalis* strain that reduced the amount of toxin present in the sample by 80%. The highest efficiency towards ochratoxin A (97%) was demonstrated by a *Lactobacillus acidophilus* strain. On the other hand, Zheng et al. [122] used, in their study, a *Lacticaseibacillus casei* (*Lactobacillus casei*) strain to test the optimum conditions for patulin neutralization. The results confirmed the very good capabilities of L. casei to eliminate the toxin from the environment, and they also demonstrated that the temperature of 30 °C and pH of 5.0 are most favorable for the process. In addition, it was determined that, in the case of patulin, live cells exhibit a considerably higher efficiency in neutralizing patulin as compared with thermally inactivated cells. Therefore, it can be concluded that the mechanism of toxin removal is not only linked to the temperature or pH of the environment but also to the type of mycotoxin being neutralized. 

Numerous studies show that LAB can inactivate aflatoxins [123,124], zearalenone [125,126], deoxynivalenol [125,127] and fumonisins [128]. Niderkorn et al. [125] tested the possibility of LAB to bind mycotoxins biosynthesized by Fusarium fungi. Fumonisin B2 was most efficiently removed from the environment, followed by zearalenone, deoxynivalenol and fumonisin B1. The study of Cvek et al. [129] utilized *Lactiplantibacillus plantarum* (*Lactobacillus plantarum*) and *Lacticaseibacillus rhamnosus* (*Lactobacillus rhamnosus*) to neutralize zearalenone under in vitro conditions. The study demonstrated the capability of these strains for the adhesion of the mycotoxin to the cell wall at 37 °C within 72 h. It was observed that the higher the bacterial cell concentration, the higher the efficiency of the process. Within the first hours of incubation, 95–97% of zearalenone was bound to the cell wall of the bacteria; yet, during the subsequent hours, the percentage was reduced due to the re-release of the toxin back to the environment, which confirms that the adhesion process is reversible with time. In the case of fumonisins, numerous reports suggested that the neutralization of these toxins by lactic strains occurs mainly through adhesion, and the process intensity is strictly linked to the species’ cell wall structure [130,131,132]. Similarly, aflatoxin binding by LAB can be directly linked to the occurrence of peptidoglycans and polysaccharide in the cell wall. Thus, future research should be focused on the assessment of differences in the structure of cell walls between LAB species in order to select the most appropriate strain to remove the specific mycotoxin from the environment [133,134].

## 9. Conclusions

Biopreservation may determine the biological alternative for chemical and physical methods of food preservation, which are generally considered as negative for the quality of the product and, in some cases, negative for health. Biopreservation based on the use of LAB and their metabolites may be associated with an increase in food safety as well as other benefits for human health, considering their ability to improve nutritional value by producing some vitamins, organic acids and other compounds. LAB show antibacterial and antifungal activity. However, there is a necessity to investigate the activity of LAB against foodborne pathogens in situ to establish the most effective method of application in the food model. To achieve this aim, there is a need to understand the influence of environmental factors such as pH, temperature, food matrices and the presence of various interfering substances on the survival of some strains of LAB and their activity. In addition, LAB may detoxify second metabolites of filamentous fungi using different mechanisms, including bioabsorption, biotransformation and bioadhesion. Most data suggest that the main mechanism of mycotoxin reduction is a binding to the cell wall, but the ability of bacteriocins production as well as other metabolites should be considered as an efficient factor in mycotoxin’s neutralization process. Taking into account how serious of a problem mycotoxins are in the food chain, biological methods of degradation by lactic acid bacteria and their metabolites (bacteriocins) should be better known through future studies.

## Figures and Tables

**Table 1:** The most used chemical preservatives and examples of their negative health impact.

Chemical Food Preservatives	Type of Food	Negative Effects	References
Sulphur dioxide (E220)	Dried fruits, juices	Asthma episodes, diarrhea, nausea and other gastric effects, loss of vitamin B1	[9,10,11]
Potassium nitrate (E249)	Cured and canned meat products	May cause lower oxygen carrying capacity of blood	[9,10]
Sodium benzoate (E211)	Pickles, sauces	Suspected neurotoxicity and cancerogenic properties, aggressive asthma episodes	[9,10]
Calcium benzoate (E213)	Cereals, meat products, low sugar products	Inhibition of digestive enzyme function	[9,10]
Benzoic acid (E210)	Pickles, sauces, meat products	Possible allergic reaction	[9,11]
Sorbic acid (E200)	Beverages, cheese, pickles, fish and meat products	Possible allergic reaction	[9,11]

**Table 2:** Characteristic products obtained through lactic fermentation, listing dominant and collaborating microflora.

Fermented Foods	Main Ingredients	Dominant Microflora	Collaborators	Country	References
Kefir	Milk	*Lactobacillus*, *Lactococcus*, *Leuconostoc*, *Oenococcus*, *Pediococcus*, *Streptococcus*	Acetic acid bacteria, yeast	International	[52,53,54]
Yogurt	Milk	*Streptococcus thermophilus*, *Lactobacillus bulgaricus*	-	International	[53,55,56]
Cheese	Milk	*Lactobacillus lactis*, *Streptococccus thermophilus*, *Lactobacillus shermanii*, *Lactobacillus bulgaricus*, *Propionibacterium shermanii*	Molds (*Penicillium*)	International	[55,56,57,58]
Kimchi	Cabbage, radish, salt	*Lactobacillus*, *Leuconostoc*, *Pediococcus*, *Weissella*	Yeast	Korea	[54,58,59,60]
Sourdough	Flour, water	*Enterococcus*, *Lactobacillus*, *Lactococcus*, *Leuconostoc*, *Pediococcus*, *Streptococcus*, *Weissella*	Yeast	International	[54,58,61]
Cucumbers	Cucumbers, garlic, salt	*Enterobacter*, *Leuconostoc mesenteroides*, *Levilactobacillus brevis (Lactobacillus brevis)*, *Lactiplantibacillus plantarum (Lactobasillus plantarum)*	-	International	[62,63]
Villi	Milk	*Lactococcus lactis subsp. cremoris*, *Lactococcus lactis* subsp. *lactis*, *Leuconostoc mesenteries*	*Geotrichum candidum*	Nordic countries	[64,65]
Sauerkraut	Cabbage, salt	*Leuconostoc mesenteroides*, *Lactococcus lactis*, *Levilactobacillus brevis (Lactobacillus brevis)*, *Lactiplantibacillus plantarum (Lactobacillus plantarum)*, *Lactobacillus pentoaceticus*	-	International	[58,66,67]

**Table 3:** The antagonistic activity of selected LAB strains against yeasts and filamentous fungi in selected fermented products. Our own elaboration on the basis of Salas et al. [94].

LAB Strains	Food Field	Source of LAB	Method of Application	Inhibited Microorganism	References
*Lactobacillus harbinensis* K.V9.3.1Np, *Lacticaseibacillus rhamnosus* K.C8.3.1I (*Lactobacillus rhamnosus* K.C8.3.1I), and *Lacticaseibacillus paracasei* K.C8.3.1Hc1 (*Lactobacillus paracasei* K.C8.3.1Hcl)	yogurt	cow and goat milk	cells as adjunct culture	*Debaryomyces hansenii*, *Kluyveromyces lactis*, *Kluyveromyces marxianus*, *Penicillium brevicompactum*, *Rhodotorula mucilaginosa*, and *Yarrowia lipolytica*	[96]
*Lacticaseibacillus casei* AST18 (*Lactobacillus casei* AST18)	yogurt	chinese dairy products	cells as adjunct culture	*Penicillium* sp.	[97]
*Lactobacillus amylovorus* DSM 19280	cheddar cheese	cereal environment	cells as adjunct culture	*Penicillium expansum* and environmental molds	[98]
12 strains of *Lactiplantibacillus plantarum* (*Lactobacillus plantarum*)	cottage cheese	fresh herbs, fruits, and vegetables	cells as added to the finished product	*Penicillium commune*	[99]
*Lacticaseibacillus paracasei* DCS302 (*Lactobacillus paracasei* DCS302)	yogurt	no data	cells as adjunct culture	*Penicillium* sp. nov. DCS 1541, *Penicillium solitum*	[100]
*Lactobacillus harbinensis* K.V9.3.1Np	yogurt	cow milk	cells as adjunct culture	*Yarrowia lipolytica*	[96]
*L. rhamnosus* A238, *L. rhamnosus* A119 (2/5) The association of *L. rhamnosus* A238 with *B. animalis* subsp. *lactis* A026, and *L. rhamnosus* A119 with *B. animalis* subsp. *lactis* A026	cottage cheese	no data	cells added to the finished product	*Penicillium chrysogenum*	[101]
*Lactobacillus amylovorus* DSM19280	sourdough quinoa bread	cereal isolate	cells in sourdough	environmental molds	[102]
*Lactiplantibacillus plantarum* CRL778 (*Lactobacillus plantarum* CRL778)	wheat bread	homemade wheat dough	SL778: fermentate as ingredient	environmental molds	[103]
*Lactobacillus amylovorus* DSM19280	sourdough wheat bread	cereal isolate	cells as starter	*Fusarium culmorum*	[102]
*Lactiplantibacillus plantarum* (*Lactobacillus plantarum*) UFG 121 (only 1 in situ from best 2/88 in vitro)	oat-based product	food	cells in sourdough	*Fusarium culmorum* (only 1 tested in situ), *Penicillium chrysogenum*, *Penicillium expansum*, *Penicillium roqueforti*, and *Aspergillus flavus* (5/7 in vitro)	[104]
*Lactobacillus bulgaricus* CECT 4005, *L. plantarum* CECT 749 (active in situ 2/6), *Lactobacillus johnsonii* CECT 289, *L. rhamnosus* CECT 288, *L. ruminis* CECT 1324 *and Bifidobacterium bifidum* CECT 870T (6 active in vitro/16)	bread	no data	cells in sourdough	*Aspergillus parasiticus* (only one tested in situ) and *Penicillium expansum*	[88]
*L. delbrueckii group*, *L. alimentarius group*, *L. plantarum group*, *L. casei group*, *L. buchneri group*, *L. perolens group*, *L. sakei group*, *L. fructivorans group*, *L. reuteri group*, *L. brevis group L. rossiae*, *Leuconostoc* spp., *Pediococcus* spp., *Carnobacterium* spp., *Weissella* spp., *L. lactis* subsp. *Lactis*, *Propionibacterium* spp.	cakes and milk bread rolls	bread roll sourdough	sprayed on the Surface of product	Species of *Aspergillus*, *Penicillium*, *Cladosporium*, *Wallemia*, *Eurotium*	[95]
*Lactobacillus helveticus* KLDS 1.8701	fermented soybean milk	dairy products	cells as adjunct culture	*Penicillium sp.*	[105]
*Lactiplantibacillus plantarum* TK9 *(Lactobacillus plantarum* TK9)	citrus, apples and yogurt	chinese naturally fermented congee	cells	*Penicillium roqueforti*, *Penicillium citrinum*, *Penicillium oxalicum*, *Aspergillus fumigatus*, *Aspergillus flavus* and *Rhizopus nigricans*	[106]

Environmental molds–microorganisms that may occur indoors and outdoors as natural environments, including genera *Alternaria*, *Cladosporium*, *Botrytis*, *Epicoccum*, *Asperigillus*, *Rhizopus*, *Mucor* and *Penicillium* [107].

## Data Availability

Not applicable.
